# Secretory laccase 1 in *Bemisia tabaci* MED is involved in whitefly-plant interaction

**DOI:** 10.1038/s41598-017-03765-y

**Published:** 2017-06-15

**Authors:** Chun-Hong Yang, Jian-Yang Guo, Dong Chu, Tian-Bo Ding, Ke-Ke Wei, Deng-Fa Cheng, Fang-Hao Wan

**Affiliations:** 1 Key Laboratory for Biology of Plant Disease and Insect Pests, Institute of Plant Protection, Chinese Academy of Agricultural Sciences, Beijing, 100193 China; 2 Key Lab of Integrated Crop Pest Management of Shandong Province, College of Agronomy and Plant Protection, Qingdao Agricultural University, Qingdao, 266109 China

## Abstract

The whitefly *Bemisia tabaci* is a phloem-feeding pest that lives predominantly on herbaceous species and causes serious damage to hosts. Whitefly saliva is thought to contain proteins that modulate plant defences and facilitate feeding. A predicted secreted protein, laccase 1 (LAC1), was found in the salivary gland transcriptome of *B. tabaci* and might be existed in the watery saliva of *B. tabaci*. As LAC1 has a potential role in detoxification of secondary plant compounds in insects, we speculated that it may participate in the insect’s response to plant defences. Here, we cloned the complete cDNA of *LAC1* and found that (1) *LAC1* was highly expressed in the salivary gland (SG) and midgut; (2) *LAC1* transcript level in head (containing SG) was 2.1 times higher in plant-fed than in diet-fed whiteflies and 1.6 times higher in the head and 23.8 times higher in the midgut of whiteflies that fed on jasmonic acid (JA)-sprayed plants than on control plants; and (3) silencing *LAC1* decreased the survival rate of plant-fed whiteflies but had a marginal effect on whiteflies raised on an artificial diet. These results indicate that LAC1 enables whiteflies to overcome the chemical defences of host plants and might act as an effector in saliva.

## Introduction

Throughout the history of co-evolution, both host plants and herbivorous insects have developed a wide variety of complex defence and anti-defence strategies^1, 2^. Plants have evolved strategies to defend themselves against insect herbivory that are based on physical barriers, constitutive chemical defences, and directly and indirectly inducible defences^3^. Conversely, insects have developed sophisticated mechanisms to overcome these plant defences. Their response takes the form of changes in gene expression and the protein repertoire in cells lining the alimentary tract, the first line of defence. To survive, insect herbivores adjust their feeding habits, digestive physiology, and gene expression to optimally adapt to their hosts^4^.

The whitefly *Bemisia tabaci* (Gennadius) (Hemiptera: Aleyrodidae) is an efficient plant-feeding insect with a global distribution that causes serious damage to crops by its direct feeding or by the transmission of plant viruses^5–7^. *B. tabaci* is a complex species with at least 36 morphologically indistinguishable cryptic species^5, 8^. Among them, *B. tabaci* MED (commonly known as biotype Q) has now spread to most parts of China and has caused significant damage to host plants^8–11^.

Importantly, the whitefly can successfully and persistently feed on phloem sap from sieve elements^12^, and its saliva is thought to contain effector proteins that overcome plant defences^13–15^. Saliva injected into plant tissues has long been considered to play crucial roles in aiding penetration and nutrient ingestion and modulating plant responses, and some secreted proteins have been identified as effectors that inhibit plant defences, prevent phloem sieve-element occlusion, and otherwise promote the unique phloem feeding style^16^. Scientists have found several effectors in aphids, such as C002, Mp10, Mp55, Me10, Me23, and Armet, which play an important role in overcoming plant defences and helping the herbivore establish a population on host plants^3, 16–20^. However, there are few reports on effectors in whiteflies.

We found a predicted secreted protein, laccase 1 (benzenediol:oxygen oxidoreductase; EC 1.10.3.2, LAC1), in the salivary gland transcriptome of *B. tabaci* (data unpublished). Though several laccase isoforms have been identified in insects^21–27^, less is known about insect LAC1. LAC1 belongs to the multicopper (MCO) family^28^, it has been proposed that insect LAC1 may play an important role in metal ion metabolism, lignocellulose digestion and detoxification of secondary plant compounds^23, 26^. As LAC1 has many potential catalytic and oxidative functions, we speculated that it may participate in the insect’s response to plant defences.

The goal of this study was to elucidate the LAC1 function in *B. tabaci* and confirm its putative functions as an effector protein that modulates whitefly–plant interactions. In this study, we first cloned the complete cDNA sequence and determined the temporal and spatial expression pattern of *LAC1*. Next, to clarify whether LAC1 is related to the insect’s response to plant defences, we compared the expression differences between plant-fed and diet-fed *B. tabaci* and between jasmonic acid (JA)-sprayed plant-fed and control plant-fed *B. tabaci*. Finally, using RNAi, we examined whether the presence of LAC1 influences *B. tabaci* survival rates on tomato plants. This study helps us to understand the mechanisms by which whiteflies overcome plant defences.

## Results

### Characteristics of the *LAC1* gene and protein

The complete cDNA of *LAC1* contained an open reading frame (ORF) of 2733 bases encoding a protein of 911 amino acid residues with a predicted molecular weight of 103.03 kDa (Figure S1), the GenBank accession number of LAC1 is KY643659. SignalP predicted an N-terminal signal secretion peptide with a cleavage site between Pro^19^ and Thr^20^. No transmembrane domain was found, suggesting that LAC1 is a secreted protein. SOSUI predicted the average hydrophobicity to be −0.483388, which suggests that LAC1 is a soluble protein. The encoded protein has three typical Cu-oxidase domains in the polyphenol oxidase family, indicating that LAC1 belongs to the blue copper-containing polyphenol oxidase family.

The full-length *LAC1* also showed a high level of amino acid sequence identity with *LAC* sequences from other insects found in GenBank. An alignment of the protein sequences of 18 laccases is shown in Figure S2. The origin of the *LAC* genes and their GenBank accession numbers are listed in Table S2. Using BLAST analysis, transcripts of Cu-oxidase domains homologous to *B. tabaci* LAC1 were identified in many insects. The Cu-oxidase domains seemed to be relatively conserved among all the studied insect laccases.

### Sequence retrieval and phylogenetic analysis

**Figure 1:**
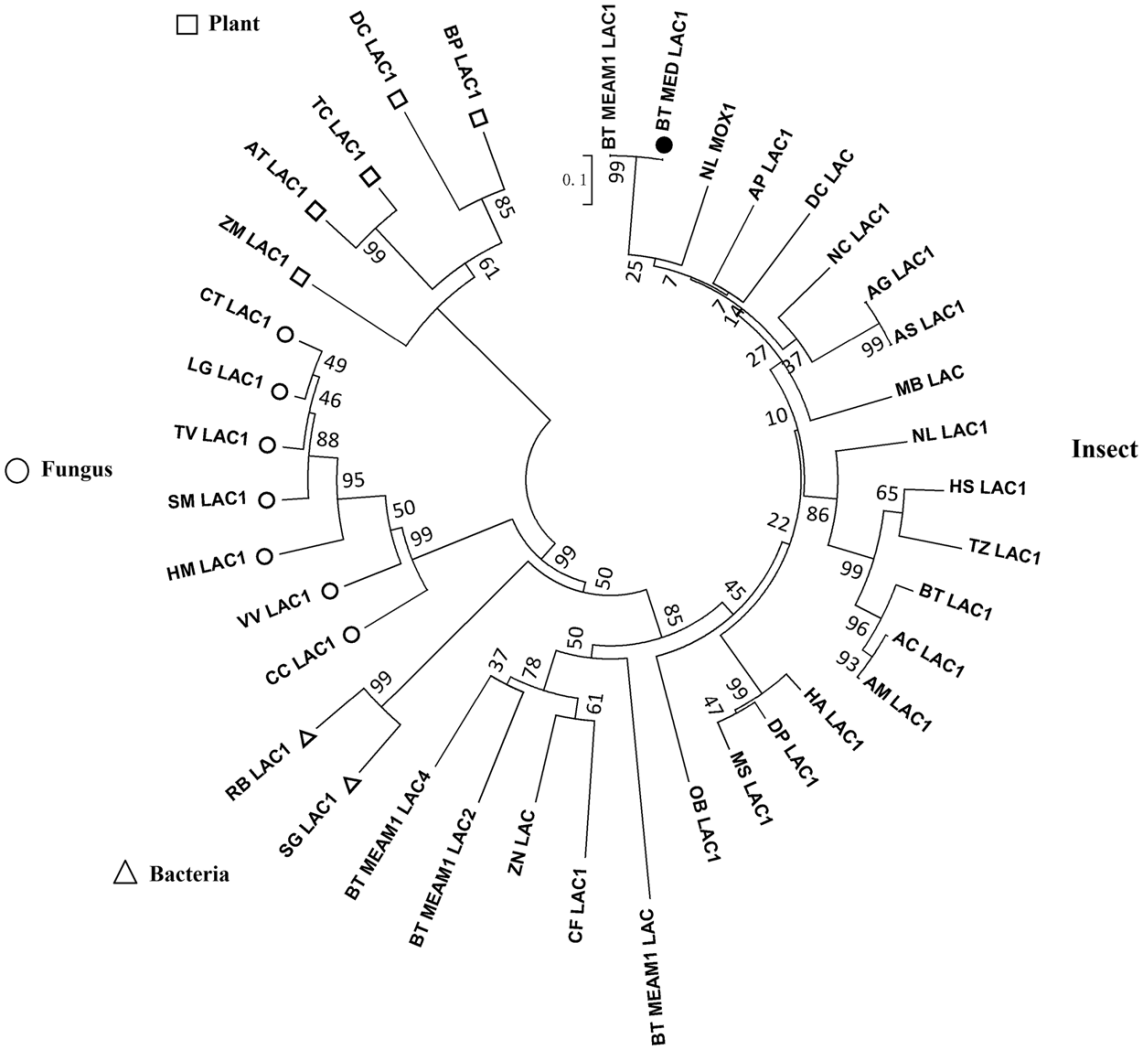
Phylogenetic trees comparing *B. tabaci* MED LAC1 and the known insect LAC genes. The phylogenetic tree was generated by MEGA 5.05 based on the neighbour-joining method according to amino acid sequences. Bootstrap support values calculated on 1000 replications are shown on the branches. The laccase sequences used to generate the tree are listed in Table S3.

Phylogenetic analyses were conducted to examine the relationships among *B. tabaci* MED LAC1 and the LAC genes of other insects, bacteria, fungus and plants (Fig. 1). The LAC genes of *B. tabaci*, *Diaphorina citri, Acyrthosiphon pisum, Nilaparvata lugens* and *Nephotettix cincticeps*, which are all Homoptera insects, are closely related. However, BT MEAM1 LAC2 and BT MEAM1 LAC4 are quite genetically distant from LAC1, indicating that LAC1 is functionally distinct from other LACs. The phylogenetic analysis also revealed that laccases from these groups (viz., insects, bacteria, fungi and plants) form independent clades consistent with their taxonomical classification.

### Temporal and spatial expression of *LAC1*

**Figure 2:**
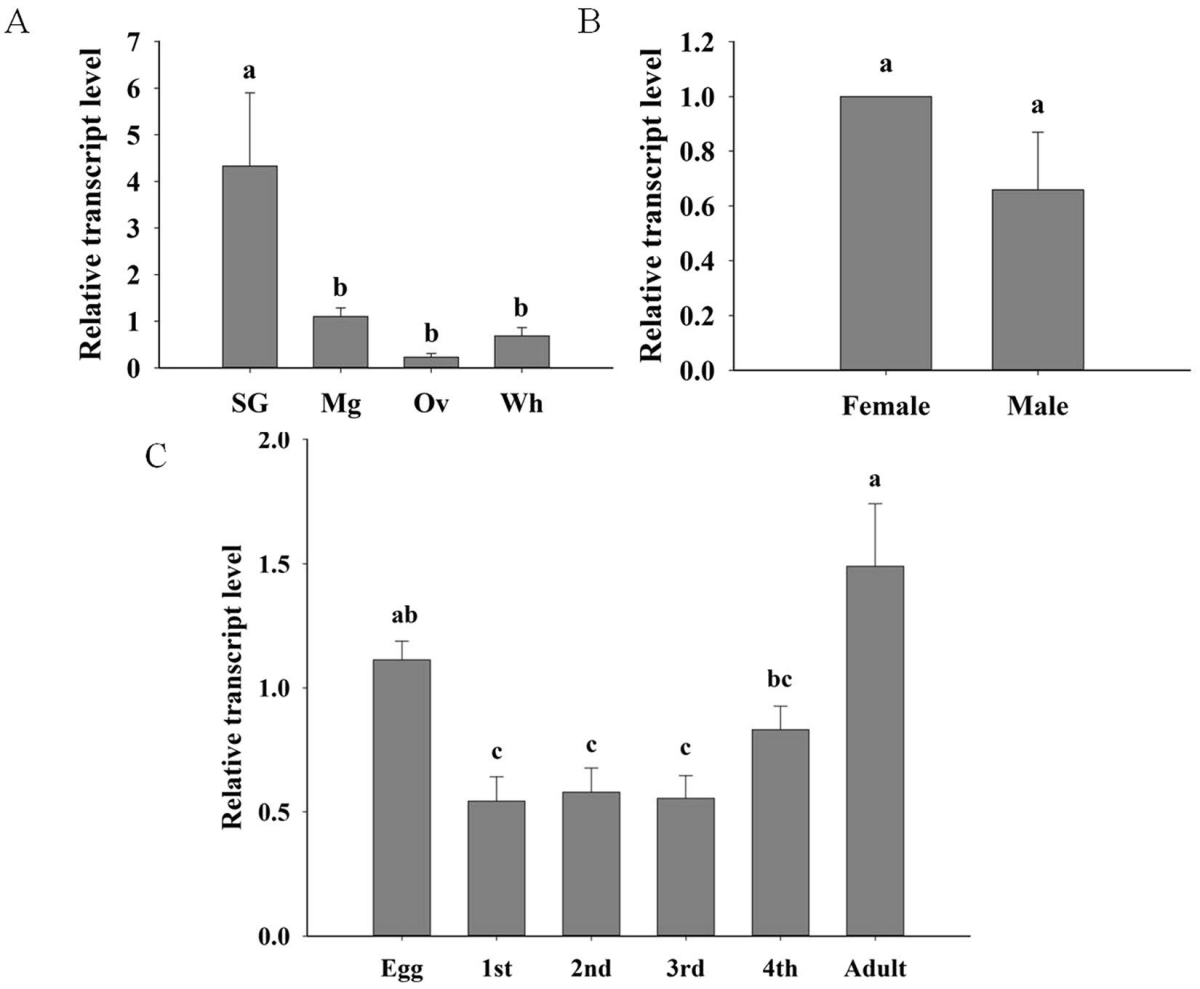
Temporal and spatial expression of *B. tabaci* LAC1 measured by real-time qPCR. (**A**) Expression in salivary gland (SG), midgut(Mg), ovary(Ov) and whole body (Wh). (**B**) Expression in female and male. (**C**) Expression in egg, larval and adult stages. Values are represented as the mean ± SEM. The letters above the columns indicate the comparison among groups evaluated, p < 0.05.

RNA from the salivary gland, midgut and ovary was analysed using real-time quantitative PCR (qPCR) to determine the transcript levels of *LAC1*. *LAC1* is most highly expressed in the salivary gland and midgut (Fig. 2A) and is expressed at a higher level in female adults than male adults (Fig. 2B). *LAC1* mRNA is expressed consistently in all stages (Fig. 2C).

### LAC was found in the watery saliva of *B. tabaci*

LAC activity was examined in *B. tabaci* fed a sucrose diet through a membrane (Table 1). ABTS is thought to be the best substrate to detect laccase^21^. ABTS only reacts with LAC at pH 5; therefore, the results measured in whitefly saliva mixed with ABTS represented the enzyme activity of LAC. This experiment demonstrated that LAC exists in *B. tabaci* watery saliva.

**Table 1:** Laccase activities in *B. tabaci* flies fed a sucrose diet.

Substrate (2 mM)	Absorbance at 420 nm		
	After feeding	Unfed control	
ABTS, pH 5	0.0447 ± 0.0004	0.0427 ± 0.0002	p = 0.0012

Two hundred *B. tabaci* adults were exposed to 200 μL of a 10% sucrose solution for 24 h, and subsequently, 50 μL of the recycled feeding liquid was analysed in a microplate reader at 28 °C for 5 min. Mean ± SEM, n = 3.

### *LAC1* expression was higher in plant-fed than diet-fed whiteflies and higher in JA-sprayed plant-fed than control plant-fed whiteflies

**Figure 3:**
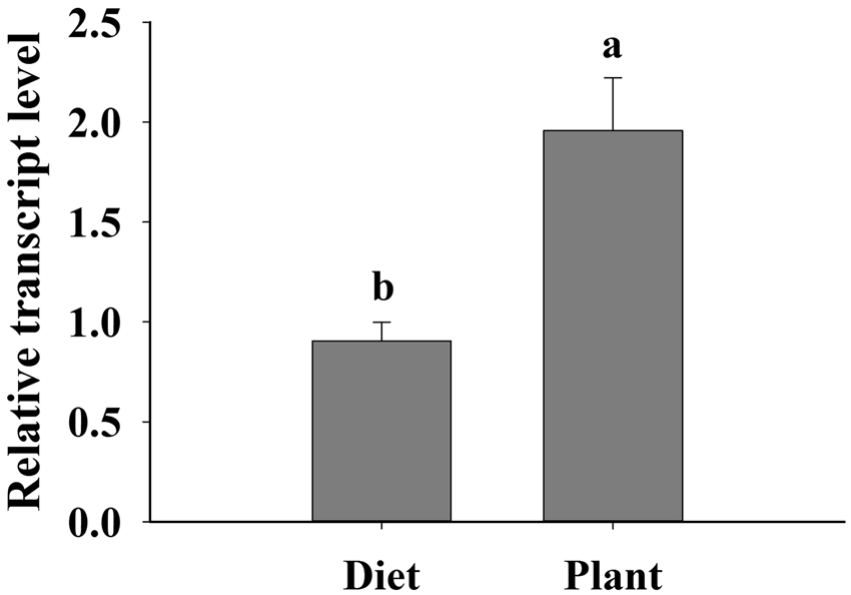
Transcript levels of LAC1 in the heads (containing the salivary glands) of diet- and plant-fed *B. tabaci*. Values are represented as the mean ± SEM. The letters above the columns indicate the comparison among groups, p < 0.05.

To explore whether *LAC1* is necessary in plant feeding, the transcript levels of *LAC1* in *B. tabaci* heads, which contain the salivary glands, were compared between plant-fed and artificial diet-fed *B. tabaci* using qPCR. The normalized level of the *LAC1* transcript was approximately 2.2-fold higher in plant-fed *B. tabaci* than in artificial diet-fed *B. tabaci* (Fig. 3). This result suggests that more *LAC1* was required by *B. tabaci* during interactions with host plants.

**Figure 4:**
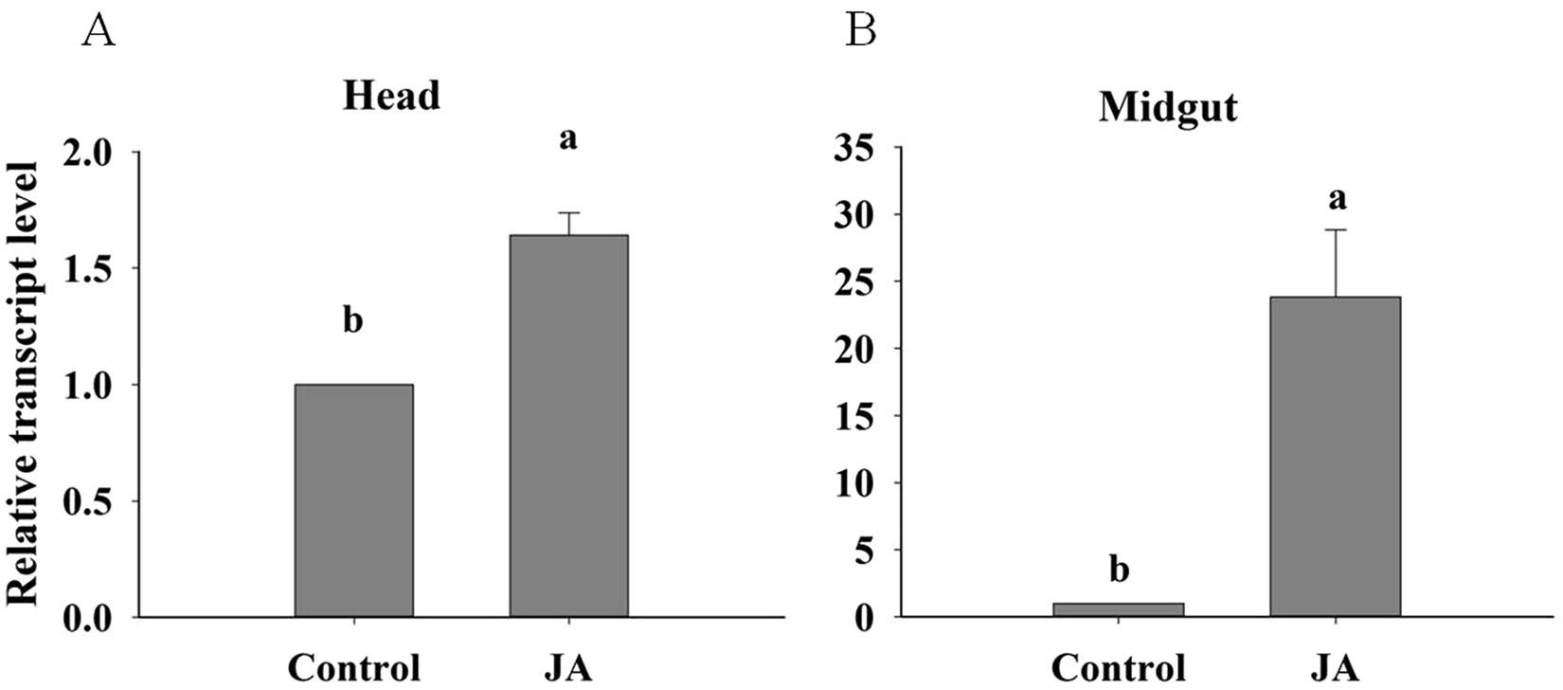
Transcript levels of LAC1 in JA-sprayed plant-fed or control plant-fed *B. tabaci*. (**A**) Expression in heads (containing the salivary glands). (**B**) Expression in the midgut. Values are represented as the mean ± SEM. The letters above the columns indicate the comparison among groups, p < 0.05.

To explore whether *LAC1* is related to the insect’s response to plant defences, we compared the difference in *LAC1* expression between JA-sprayed plant-fed and control plant-fed whiteflies. Tomato plant defences can be induced by spraying them with exogenous JA^29, 30^. When *B. tabaci* flies fed on tomato plants that were sprayed with JA for 2 days, the LAC1 expression in the head increased approximately 1.64-fold (Fig. 4A), and the *LAC1* expression in the midgut increased approximately 23.81-fold (Fig. 4B). These results show that *LAC1* is required to counteract the defence reaction of plants.

### *LAC1* is indispensable for *B. tabaci* survival on tomato plants

**Figure 5:**
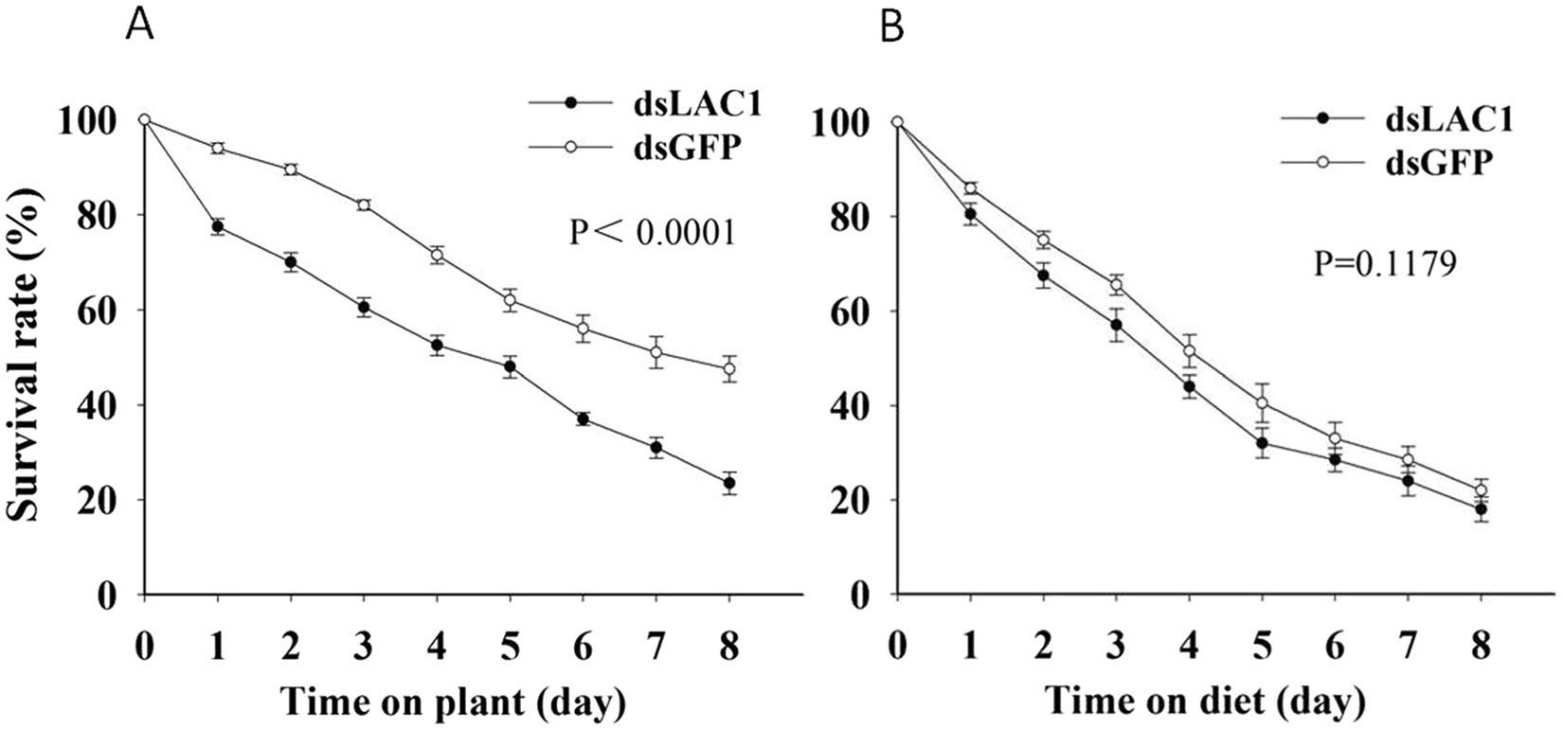
Knocking down *LAC1* decreases the survival rates of *B. tabaci* adults. (**A** and **B**) Mean survival rates of *B. tabaci* adults that were fed dsRNA against *LAC1* (*dsLAC1*) or *GFP* (*dsGFP*) and then fed on tomato plants (**A**) or an artificial diet (**B**). P values of the difference between the curves of the interference and control groups are indicated, p < 0.05.

**Figure 6:**
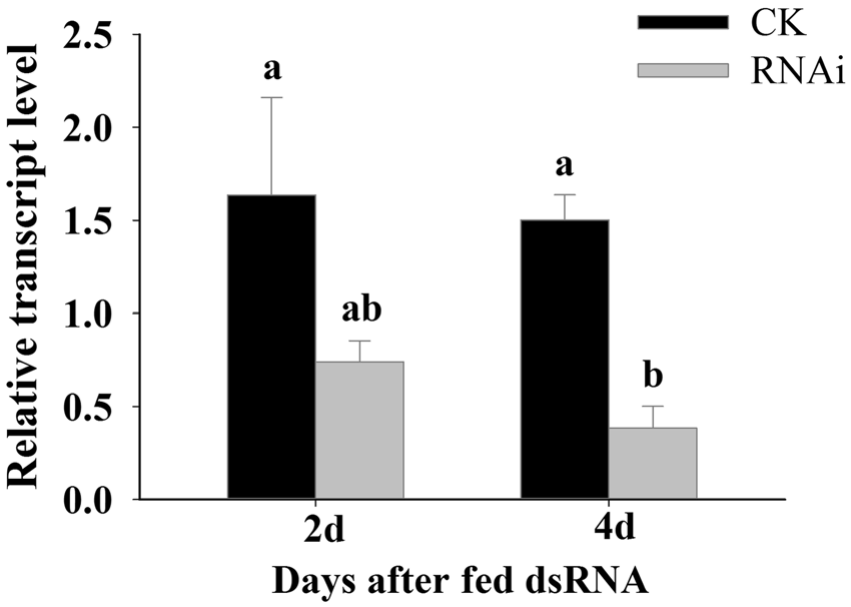
Transcript levels of *B. tabaci* LAC1 after dsRNA feeding. Values are represented as the mean ± SEM. The letters above the columns indicate the comparison among groups evaluated by an ANOVA using SAS 8.1, p < 0.05.

To test whether the presence of *LAC1* influences *B. tabaci* survival rates on tomato plants and whether this influence is related to the effect of *LAC1* on the ability of *B. tabaci* to reach the phloem, we compared the performance of *B. tabaci* adults on different food matrices. *LAC1* silencing generally reduced the survival rate (Fig. 5), and the effect was most pronounced on tomato plants. When *B. tabaci* was fed *LAC1* dsRNA, the *LAC1* transcript level in the *B. tabaci* whole bodies was reduced by approximately 55% and 74% at 2 and 4 days after feeding, respectively (Fig. 6). *B. tabaci* flies were fed *LAC1* dsRNA for 2 days and then moved onto *Lycopersicon esculentum* or fed an artificial diet. Compared with a control *GFP* dsRNA, the *LAC1* dsRNA significantly reduced the survival rates of *B. tabaci* that fed on host plants (P < 0.001) (Fig. 5A) but had a smaller influence on *B. tabaci* given an artificial diet (P = 0.1179) (Fig. 5B). The survival rate of *B. tabaci* adults with silenced *LAC1* was much higher in insects raised on an artificial diet than in those raised on plants. These results demonstrate that LAC1 contributes to the survival rate of *B. tabaci* adults raised on tomato plants.

## Discussion

Though there have been some reports on insect laccases, the physiological functions of insect LAC1 have not been established. Our experiments demonstrate that LAC1 is required for *B. tabaci* survival on tomato plants and is important in enabling whiteflies to overcome the chemical defences of host plants.

### Identification of LAC1

Currently, information on the possible LAC1 substrates in the whitefly is lacking, and LAC1 in *B. tabaci* may perform more than one function. The physiological substrates of LACs that are hydrolysed *in vivo* have not been fully elucidated in insects. To perform its catalytic function, laccase depends on Cu atoms that are distributed at three different copper centres. These copper centres in laccases are categorized into three groups: Type-1 (T1), Type-2 (T2) and Type-3 (T3)^31^. The laccase genes typically have three to four of these copper domains^32^, the structure of which can vary greatly between different organisms. This extensive structural variability results in a corresponding variation in catalytic properties and oxidative function^33–36^, such as their involvement in lignin biosynthesis (in plants) as well as lignin degradation (in fungi and bacteria)^31^. Laccases catalyse the oxidation of a wide array of substrates during four-electron transfer processes while reducing O_2_ to water^37^. We found that LAC1 also has three different copper domains: T1, T2 and T3. This structural characteristic demonstrates that LAC1 belongs to the laccase family and has catalytic properties as well as oxidative function. In contrast to most enzymes, which catalyse substrate-specific reactions, laccases oxidize a wide range of compounds that vary from di-, poly-, and substituted phenols to diamines, aromatic amines, and benzenethiols^31, 38^. As laccases have so many kinds of substrates, LAC1 in *B. tabaci* may perform more than one function *in vivo*.

Phylogenetic analyses comparing the *B. tabaci* MED LAC1 protein sequence to 37 other LACs from insects, fungi, bacteria, and plants showed that the *B. tabaci* laccases are evolutionarily unique and distinct from other insect LAC2 proteins with known or suspected roles in integument formation^23, 26, 39^. According to the phylogenetic analysis, *B. tabaci* LAC1 is most similar to *N. lugens* MOX1 (AKN21378.1), *D. citri* LAC1 (XP_008487811.1), *N. cincticeps* LAC1 (BAJ06132.1) and *A. pisum* LAC1 (XP_001948070.1) (Fig. 1). This result illustrated that laccases of plant-sucking insects may have similar physiological functions and be evolutionarily related.

ABTS is specifically oxidized by laccase^40^. We observed a significant difference in laccase activity between saliva and the control solution, indicating that the ABTS-oxidizing enzyme is laccase. So far, LAC1 is the only laccase that has been identified in insect saliva; for example, LAC1 has been shown to be expressed in the salivary glands and secreted into the saliva of green rice leafhoppers and termites^21–23^. Therefore, we concluded that the laccase we found in *B. tabaci* saliva might be LAC1.

### Functional analysis

The temporal and spatial expression of *LAC1* was determined and indicated that *LAC1* mRNA is expressed consistently in all stages and is most highly expressed in the salivary gland and midgut. This result suggested that as a secretory protein, LAC1 may be produced by the salivary gland and midgut epithelial cells and secreted into the salivary channel and lumen, respectively. Our results indicate that *LAC1 is* expressed throughout development and growth and plays a crucial role in biological processes. Interestingly, *LAC1* was also found to be expressed in the ovaries of *B. tabaci*, and a higher expression level was observed in female adults than in male adults. This suggests that *LAC1* may have other biological functions. For instance, LAC1 produced in the ovaries may be secreted into the plants by the ovipositor of the female adult to counteract plant responses and assist in egg deposition. Further research is necessary to elucidate this role of LAC1.

Hattori *et al*. (2005)^21^ detected LAC1 in the watery saliva and salivary glands of *N. cincticeps* and proposed that one of the functions of LAC1 is the promotion of rapid oxidative gelling of the stylet sheath by the quinine tanning reaction, as laccase is hypothesized to be involved in insect cuticle sclerotization^24, 25, 41^. As a phloem-feeding insect like *N. cincticeps*, *B. tabaci* may also use LAC1 to promote the oxidative gelling of the stylet sheath. Huang *et al*. (2016)^42^ found the survival rate of *dssalivap-3* treated insect on plant was decreased, and salivap-3 is involved in the formation of the salivary sheath. Moreover, the different survival rate of *dsNlShp* treated insect on plant and artificial diet was also observed in salivary sheath-deficient brown planthoppers, similar with the result of *dsLAC1* treatment. The NlShp acts as the main component of both salivary sheath and salivary flange^43^. So we speculate that LAC1 is synthesized in primary salivary glands and transported out through the salivary ducts, promoting rapid oxidative gelling of the stylet sheath and salivary flange to harden.

LAC1, as a secretory protein, is released into the salivary channel and then secreted via the mouth parts into plant tissues. Laccase catalyses the oxidation of phenolic lignin units as well as a wide variety of phenolic compounds and aromatic amines^38^. Penetrating plant tissues intracellularly, the stylets of whiteflies rupture the walls of the epidermal and mesophyll cells to access the phloem. The LAC1 ejected along with the watery saliva may play a role in the oxidation of phenolic compounds in plant tissues. This function helps *B. tabaci* successfully feed by inhibiting the defence reaction of plants.

When comparing the *LAC1* expression level in whiteflies fed either plants or an artificial diet, we found that whiteflies required additional *LAC1* when they fed on plants than on an artificial diet. Furthermore, the *LAC1* transcript level was several times higher in the salivary gland and midgut when the whiteflies fed on *L. esculentum* sprayed with JA than when they fed on control plants. Exogenous JA can activate plant defence genes either via the octadecanoid pathway or by acting directly on the genes^29, 30, 44^. Activation of defence genes leads to metabolic reconfiguration. Secondary metabolites such as phenols, terpenoids and alkaloids involved in plant defence response are released into phloem sap and transported throughout the plant^45, 46^, and the phloem sap is then consumed by *B. tabaci*. Coy *et al*. (2010)^23^ found that laccases are secreted from the salivary gland into the *Reticulitermes flavipes* termite midgut and play a role in lignocellulose-related phenol oxidation in the termite gut. We found that *LAC1* was expressed both in the salivary gland and midgut, so we hypothesize that, similar to *R. flavipes, B. tabaci* LAC1 is secreted from the salivary gland into the midgut and plays a role in detoxifying phenolic compounds and other toxic compounds. Furthermore, Liu *et al*. (2016)^47^ found that engineered pseudomonas putida cells expressing surface-immobilized laccases could completely eliminate chlorpyrifos via direct biodegradation. LAC1 may also help *B. tabaci* detoxify pesticides consumed with the plant phloem sap.

Feeding whiteflies with either *LAC1* dsRNA or a *GFP* control generally resulted in a reduced survival rate on plants or an artificial diet; this phenomenon may have been caused by man-made mechanical damage when transferring the insects. However, because the treated and control insects were handled in the same manner, this damage did not affect the results of the experiments. *LAC1* silencing generally reduced the survival rate, and the effect was most pronounced on tomato plants. This result demonstrates that LAC1 contributes to the survival of *B. tabaci* adults raised on tomato plants.

In conclusion, we speculate that LAC1 in the whitefly saliva, salivary gland and midgut may enable the insect to overcome the chemical defences of host plants. LAC1 might be an effector involved in modulating *B. tabaci* feeding and survival on plants. This study suggests a potentially important role for LAC1 in determining the patterns of whitefly–plant relationships through detoxifying defensive phytochemicals.

## Materials and Methods

### Insects and host plants

*B. tabaci* MED whiteflies were maintained on tomato plants (*L. esculentum*, cv. Zhongza 9) in separate greenhouses at 25 ± 1 °C with a photoperiod of 14:10 h light:dark and 60–70% relative humidity. Tomato plants were individually grown in plastic pots 9 cm in diameter in greenhouses under the same conditions as previously mentioned. Tomato plants at the five-to-six true leaf stage were used in these experiments.

### Detection of LAC 1 activity in *B. tabaci* watery saliva

Active adult *B. tabaci* raised on the tomato plants were chosen for the experiment. The method of Peng *et al*. (2013)^48^ was used with some small modifications. Briefly, the whiteflies were collected and starved for 4 h to eliminate the possible effect of the ingested plant laccase. Then, we randomly placed 200 *B. tabaci* adults into a glass tube with two transparent ends (3 cm in diameter and 8 cm in height), and the top of each glass tube was covered with two pieces of parafilm for artificial feeding. After that, we injected 200 μL of artificial diet solution into the space between the two pieces of parafilm. The solution was a dissolved mixture of 10% sucrose plus 10 mM MES (2-morpholinoethanesulfonic acid) in distilled water. A whitefly-free tube was used as the control.

After 24 h, a disposable syringe was used to recover the diet solution, which now contained the saliva of the whiteflies (hereafter referred to as recycled liquid). Care was taken to avoid breaking the basement membrane in the aspiration process. The control treatment was also recovered in a similar manner. We dispensed 50 μL of the recycled liquid into a 96-well microplate (Eppendorf, China) and then added 150 μL of ABTS (3-benzothiazole ethyl benzene sulfonic acid) substrate solution (see details for preparation below) and mixed it with the recycled liquid by pipetting up and down. The kinetics of the 200 μL ABTS substrate-enzyme was determined by measuring the absorbance of light at 240 nm, and the measurements were taken 0 min and 5 min after the solutions were mixed together. Each treatment had three analytical replicates on the microplate. The reaction was carried out at 28 °C, and the enzyme activity was calculated as the change in light absorbance per litre of solution per unit of time (OD_240nm_·min^−1^·L^−1^).

The ABTS substrate solution was prepared as follows: (1) A 100 mM NaAc (sodium acetate) solution was made by dissolving 0.8203 g of NaAc in 100 mL of distilled water. (2) A 2.0 mM ABTS solution (pH 5.0) was made by dissolving 0.1097 g of ABTS in 100 mL of the 100 mM NaAc solution. The pH was adjusted to 5, and the solution was stored in a brown bottle^49^.

### Cloning of the *B. tabaci LAC1* transcript

Total RNA was extracted from *B. tabaci* using Trizol (Invitrogen, Carlsbad, CA, USA) and then reverse transcribed into cDNA with a PrimeScript® 1st Strand cDNA Synthesis Kit (TaKaRa, Japan). The template for rapid amplification of cDNA ends (RACE) was synthesized using the Clontech SMARTer^TM^ RACE cDNA Amplification Kit (TaKaRa, Japan).

Degenerate PCR and nested PCR were performed in a TP600 Thermal Cycler (TaKaRa, Japan). The reactions were catalysed using Ex Taq^TM^ and Tks Gflex^TM^ (TaKaRa, Japan). The PCR program consisted of an initial denaturation for 2 min at 98 °C, followed by 35 cycles of 94 °C for 30 s, 55–60 °C (based on the primer annealing temperatures) for 30 s, 72 °C for 1–2 min and a final extension at 72 °C for 10 min. The sequences of the primers designed to obtain the full-length *LAC1* are shown in Table 1.

The amplified PCR fragments were gel purified with the QIAquick Gel Extraction Kit (Qiagen, Germany) and then ligated into the pClone007 vector (Beijing Tsingke Biotech Co., Ltd., China) and transferred into DH5α cells for sequencing.

### Sequence retrieval and phylogenetic analysis

Protein sequences were deduced from the sequenced ORF finder (https://www.ncbi.nlm.nih.gov/orffinder/) and analysed using the SignalP (www.cbs.dtu.dk/services/SignalP) and SOSUI (http://bp.nuap.nagoya-u.ac.jp/sosui) servers, which identify the signal peptide and predict membrane proteins, respectively. The molecular weight and isoelectric points of the deduced protein sequences were calculated by the ExPASy Proteomics Server (http://cn.expasy.org/tools/pi_tool.html). Transmembrane helices were analysed using TMHMM v.2.0 (http://www.cbs.dtu.dk/services/TMHMM-2.0).

LAC sequences of *B. tabaci* MEAM1 were found in the whitefly genome database (http://www.whiteflygenomics.org/cgi-bin/bta/index.cgi). Homologous proteins from other species were identified using the NCBI BlastP software NCBI (http://blast.ncbi.nlm.nih.gov/Blast.cgi) and were aligned using DNAMAN6.0. (see Table S2 for accession numbers and related information).

The *B. tabaci* LAC1 sequence was used as a query in BLASTp searches to identify similar laccases in the GenBank database. A phylogenetic tree was constructed with 37 laccase sequences (see Table S3 for accession numbers and related information) using the neighbour-joining method with a matrix of pair-wise distances estimated by a poisson model for amino acid sequences through the MEGA 5.05 software. Bootstrap values were calculated on 1000 replications.

### qPCR analysis of *LAC1* expression patterns in different tissues and developmental stages

Three tissues (salivary glands, midgut and ovaries) were collected from approximately 200 *B. tabaci* adults for RNA extraction. Three replicates for each tissue were prepared. RNA was also isolated from the first to fourth instar nymphs and from adult *B. tabaci* flies. Three replicates and 20 individuals per replicate were prepared for each developmental stage. RNA extracted from the whole insect body was used as a reference. qPCR was performed to quantify the transcript levels of *LAC1* in the three tissues and in various developmental stages. The amplification conditions consisted of an initial denaturation at 95 °C for 30 s, followed by 40 cycles of denaturation at 95 °C for 5 s and 60 °C for 30 s. The reactions were performed on a qTOWER 2.2 Real-Time Thermal Cycler (Analytikjena, Germany). The Qlac-qF, Qlac-qR and *β*-actin qPCR primer sequences can be found in Table S1. Differences in transcript levels were analysed using a one-way ANOVA for multiple comparisons with SAS 8.1 software. The results were presented as the mean ± SEM. The relative expression levels were calculated by the 2^−ΔΔCt^ method.

### Comparison of *LAC1* transcripts in diet- and plant-fed *B. tabaci*

Sixty 0-day-old (within 1 h after eclosion) *B. tabaci* adults were placed on *L. lycopersicum* Mill plants for 96 h of feeding. Another group was fed an artificial diet (20% sucrose in distilled water) between two layers of parafilm stretched over the top of a glass tube (3 cm in diameter and 8 cm in height). The transcript levels of LAC1 in the heads (containing the salivary glands) of diet- and plant-fed *B. tabaci* were compared using qPCR. Twenty *B. tabaci* heads were included in each replicate, and three replicates were prepared. The values were reported as the mean ± SEM, and the differences were evaluated with a t-test using SAS 8.1.

### Comparison of *LAC1* transcripts in exogenous JA-sprayed plant-fed and control plant-fed *B. tabaci*

The JA was first dissolved in 1 mL of ethanol, and then distilled water was added to obtain a JA concentration of 1 mM. The JA solution was sprayed on tomato leaves. Next, 0-day-old *B. tabaci* adults were collected and transferred onto the plants 48 h after the JA spraying. Tomato plants sprayed with distilled water with 1 mL of ethanol were used as the control. The head and midgut were collected from approximately 200 *B. tabaci* adults for RNA extraction. Three replicates for each tissue were prepared. Differences were evaluated by a t-test using SAS 8.1.

### Gene interference by feeding double-stranded RNA (dsRNA) and survival curve analysis

PCR primers with T7 promoter sequences were used to prepare the dsRNA of the LAC1 gene. Two primers for the specific amplification of *dsLAC1*, LAC1-dsRNA-F and LAC1-dsRNA-R, were designed (Table S1). A dsRNA for green fluorescent protein (GFP) was amplified using the primers GFP-dsRNA-F and GFP-dsRNA-R and used as a negative control (Table S1). dsRNA was generated and purified using a TranscriptAid T7 High Yield Transcription Kit (Thermo Scientific, USA) following the manufacturer’s protocols. The dsRNA was resuspended in RNase-free water and analysed by spectrophotometric quantification and agarose gel electrophoresis. The dsRNA was stored at −80 °C until further use.

Zero-day-old *B. tabaci* adults were fed a diet containing dsRNA diluted to 100 ng·μL^−1^ in an RNase-free 20% sucrose solution for 48 h. Two hundred 0-day-old *B. tabaci* adult females were collected and placed in a glass tube (3 cm in diameter, 5 cm high); this was repeated with 200 males. The tube opening was covered with two layers of parafilm, and 500 μL of the dsRNA solution was injected into the gap between the two layers. The other end of the tube was covered with gauze for ventilation. Ten groups of *B. tabaci* flies, with 20 individuals (10 females and 10 males) in each group, were fed and subsequently reared on *L. esculentum* plants or on an artificial diet for 8 days.

Three groups of *B. tabaci* flies, with 20 individuals in each group, were collected on the second and fourth days after dsRNA feeding to test the inhibition efficiency of *LAC1* transcription in *B. tabaci* whole bodies.

The survival rate of whiteflies that fed on plants or an artificial diet was recorded every 24 h after dsRNA feeding and were reported as the mean ± SEM. The survival curves of the RNAi and control groups on a plant or artificial diet were statistically compared using the log-rank (Mantel-Cox) test.

## Electronic supplementary material

Supplementary Information
