# Supplementary material for: Secretory laccase 1 in Bemisia tabaci MED is involved in whitefly-plant interaction
Source: Sci Rep. 2017 Jun 15;7:3623. doi: 10.1038/s41598-017-03765-y (PMC5472608; doi:10.1038/s41598-017-03765-y)
Supplement: Supplementary file 1 — Supplementary Information [file 41598_2017_3765_MOESM1_ESM.pdf]

Secretory laccase 1 in *Bemisia tabaci* MED is involved in whitefly-plant interaction

Chun-Hong Yang[1], Jian-Yang Guo[1], Dong Chu[2], Tian-Bo Ding[2], Ke-Ke Wei[2], Deng-Fa Cheng[1*], Fang-Hao Wan[1,2*]

Figure S1. Nucleotide and deduced amino acid sequences of *LAC1* cDNA from *Bemisia tabaci* MED.

```
                10        20        30        40        50        60        70        80        90
1     ATGACAAACGACTCTTCCGACCTGTCCGTTAAGGTCAAATGGAAAAGTATGGAGGGAGTTAGCACAAAATGGATTACACTTTGCCTCCTG
1      M  T  N  D  S  S  D  L  S  V  K  V  K  W  K  S  M  E  G  V  S  T  K  W  I  T  L  C  L  L
               100       110       120       130       140       150       160       170       180
91    CAACTAGCAGGCTCTGTCCGTTCAATAGAGATGGATTTTCATCCATGTGATAGAAATTGCGTAGCTGGCGCTCCTGCCAGATGGTGTCAG
31     Q  L  A  G  S  V  R  S  I  E  M  D  F  H  P  C  D  R  N  C  V  A  G  A  P  A  R  W  C  Q
               190       200       210       220       230       240       250       260       270
181   TACGATTTTCACGTCCAATGGTACGAAAGTATGAGCAAGGCTTGCTACAACTGTCCGAGAAATATTACAGACTGTTCTCGCCCGGAATGC
61     Y  D  F  H  V  Q  W  Y  E  S  M  S  K  A  C  Y  N  C  P  R  N  I  T  D  C  S  R  P  E  C
               280       290       300       310       320       330       340       350       360
271   ATACCGTTAGATGGGGTTGAGAGACCAATCGTCACAGTCAATAAAATATTCCCGGGTCCTATGGTCCAGGCGTGTGTAGGTGATATGGTG
91     I  P  L  D  G  V  E  R  P  I  V  T  V  N  K  I  F  P  G  P  M  V  Q  A  C  V  G  D  M  V
               370       380       390       400       410       420       430       440       450
361   GAAATTATCGTGCATAATCACTTGCCGGAGGAAACAACGAGTATCCACTGGCATGGCCTTCACCAAAGGGAGTCCCCTTACATGGACGGC
121    E  I  I  V  H  N  H  L  P  E  E  T  T  S  I  H  W  H  G  L  H  Q  R  E  S  P  Y  M  D  G
               460       470       480       490       500       510       520       530       540
451   GTCCCTTTCGTTACCCAGTGCCCCATTCAGCCAAGATCTCGATTTAAGTACGTTTTCCTCGCCTCCACCCCTGGCACTCATTTCTGGCAC
151    V  P  F  V  T  Q  C  P  I  Q  P  R  S  R  F  K  Y  V  F  L  A  S  T  P  G  T  H  F  W  H
               550       560       570       580       590       600       610       620       630
541   TCGCACAGTGGATCTCAAAGAGGGGACGGCATGTTCGGGGGTCTGATCATCCGACGTCCGAGGAGTGAGGACCCGCACAGGAGGCTCTAC
181    S  H  S  G  S  Q  R  G  D  G  M  F  G  G  L  I  I  R  R  P  R  S  E  D  P  H  R  R  L  Y
               640       650       660       670       680       690       700       710       720
631   GACCACGACCTGCCCGAACACGTCATGACCGTCATGGACTGGCATCACCAGATGGGAACGGCCACTTTCCTCGAGCACCACCACAGCAAC
211    D  H  D  L  P  E  H  V  M  T  V  M  D  W  H  H  Q  M  G  T  A  T  F  L  E  H  H  H  S  N
               730       740       750       760       770       780       790       800       810
721   GGCACCAACAAGCCCAACAACATCCTCGTCAACGGCAAAGGACGCTACAAGAGTGGTAACGCTCACGAGGCCCTCAAGACGCCTCTTGCG
241    G  T  N  K  P  N  N  I  L  V  N  G  K  G  R  Y  K  S  G  N  A  H  E  A  L  K  T  P  L  A
               820       830       840       850       860       870       880       890       900
811   ATTTTCAACGTGAAAAAAGGTGAAAAATACAGATTCCGATTGATAAATGCAGGATTCCTCAACTGCCCAATCGAAATGTCTATTGATAAT
271    I  F  N  V  K  K  G  E  K  Y  R  F  R  L  I  N  A  G  F  L  N  C  P  I  E  M  S  I  D  N
               910       920       930       940       950       960       970       980       990
901   CACACAATCACTGTCATCAATAGCGACGGCGGTGACATCGAACCGGAAGAAGCAACCTCCCTGGTCAGCTACGCAGGTGAGCGATGGGAT
301    H  T  I  T  V  I  N  S  D  G  G  D  I  E  P  E  E  A  T  S  L  V  S  Y  A  G  E  R  W  D
              1000      1010      1020      1030      1040      1050      1060      1070      1080
991   TTCACTGTCCACCCCAGCGGTGAAGTGAAGAACTACTGGATCCGGTACCGCGGCTTGATGGATTGCGATCAAAGATTCACGTCTGCATAC
331    F  T  V  H  P  S  G  E  V  K  N  Y  W  I  R  Y  R  G  L  M  D  C  D  Q  R  F  T  S  A  Y
              1090      1100      1110      1120      1130      1140      1150      1160      1170
1081  CAAGTTGCTATCTTGCATTATGAGGGCGCACCCGATCCCACCGTCGAGGAGCCGGAAGGACAGGTTGTTTACAGCAACAGCTTCAATCCT
```

361 Q V A I L H Y E G A P D P T V E E P E G Q V V Y S N S F N P

1180 1190 1200 1210 1220 1230 1240 1250 1260

1171 GGGATGAAAATAAATCCACTGAACGAGGGATCAGAAGATAATGGAACGCTGAACATTGCAGAGCTGAGGTCGATGGAACCGCTATCGGAG

391 G M K I N P L N E G S E D N G T L N I A E L R S M E P L S E

1270 1280 1290 1300 1310 1320 1330 1340 1350

1261 AAGGATCCTTCACTCAAGGATACCGCCGACTTTACTCATTACTTAGCCTACGACTTTTACCGTATAGACAATCCTCACTACCACAAGAAA

421 K D P S L K D T A D F T H Y L A Y D F Y R I D N P H Y H K K

1360 1370 1380 1390 1400 1410 1420 1430 1440

1351 AACCTTTATGGATTCAACAGTGTGATTGAAAAGAAACGTGTGCTGACACCGCAAATCAATCATATTTCCATGAAGTTGCCGTCGTTTCCT

451 N L Y G F N S V I E K K R V L T P Q I N H I S M K L P S F P

1450 1460 1470 1480 1490 1500 1510 1520 1530

1441 CTCCTTCCTCAAAGAGATGAAATCAAGGCAGACACCTTTTGCGAACCTCATAAAATTCGAAACTGCTCAAGTGAATACTGCGAGTGCACT

481 L L P Q R D E I K A D T F C E P H K I R N C S S E Y C E C T

1540 1550 1560 1570 1580 1590 1600 1610 1620

1531 CACCACATTAAAGTTCCTTTTTCTAGCGTCGTCGAATTAGTTTTAGTAGATGAAGGTTTTGCGTATGATGCAAATCACCCCTTCCATTTG

511 H H I K V P F S S V V E L V L V D E G F A Y D A N H P F H L

1630 1640 1650 1660 1670 1680 1690 1700 1710

1621 CATGGCCATCCGTTCAGGGTTGTGGCAATGGAAAGAGTGGGCAAAAATGTGACAGTCCCACAGATTCAAGCATTGGATCGCGCTGGTTTG

541 H G H P F R V V A M E R V G K N V T V P Q I Q A L D R A G L

1720 1730 1740 1750 1760 1770 1780 1790 1800

1711 GTAAAGCGAAACCTTAAGACTGCTCCTTTGAAGGATACTGTTACCGTTCCAGATGGAGGTTACACAATAATTAGATTTCACGCGACAAAT

571 V K R N L K T A P L K D T V T V P D G G Y T I I R F H A T N

1810 1820 1830 1840 1850 1860 1870 1880 1890

1801 CCAGGTTACTGGCTATTCCACTGTCACATAGAGTTCCATGTGGAAATGGGCATGGCAGTTGTCTTCAAAATTGGCGAAGATGCAGACATG

601 P G Y W L F H C H I E F H V E M G M A V V F K I G E D A D M

1900 1910 1920 1930 1940 1950 1960 1970 1980

1891 CCACCAGTGCCCCATGGATTCCCAAAGTGCGGGGATTTTTTACCACCATTCAATGAGGAAATGGCAATGTATGATATTTACAACCTATCA

631 P P V P H G F P K C G D F L P P F N E E M A M Y D I Y N L S

1990 2000 2010 2020 2030 2040 2050 2060 2070

1981 CAAATAGCTAAGAGGCCTTCTACCTTACCAGAACAACTTCCGTCAGAATCAACCGCGAGAGAGAACCCCACAAGTCAACCCTCAACAGCG

661 Q I A K R P S T L P E Q L P S E S T A R E N P T S Q P S T A

2080 2090 2100 2110 2120 2130 2140 2150 2160

2071 GCAACAACAACCCAGTCATCGAACTCTCAATTACTGAAGAAAGGGACTTCATCGAATGTATTGAGCAGTACAATTACAGAGGAATATAAT

691 A T T T Q S S N S Q L L K K G T S S N V L S S T I T E E Y N

2170 2180 2190 2200 2210 2220 2230 2240 2250

2161 GTAGACTTATCACCTGAGACAACCACTACAGTGAATCTTCCCTTGGATCAAAATAAACAAAACCGGTACATTAGCCGGAACAGGAATGAA

721 V D L S P E T T T T V N L P L D Q N K Q N R Y I S R N R N E

2260 2270 2280 2290 2300 2310 2320 2330 2340

2251 GATAATAGGGCAGATGCAGAGGATGTCAAAATAAATGATATTTCTAGTATACTACAATTATTGCAAGGGAATATAATAAAAACTGAATAT

751 D N R A D A E D V K I N D I S S I L Q L L Q G N I I K T E Y

2350 2360 2370 2380 2390 2400 2410 2420 2430

2341 AGGTACAACCAGCACGAGGCGGCACCAGAAGTGGAGGGGACAACTATGTATAATTTACTCGATGGAAGTGATACTAATCAGCTGAGCTTC

781 R Y N Q H E A A P E V E G T T M Y N L L D G S D T N Q L S F

2440 2450 2460 2470 2480 2490 2500 2510 2520

2431 ACCAGCAAATCACCCAAAGAAAATGTAGTTCCTCCACGAAAGACCAGCTTAGATGAACTGCATTTCGCTTTTATACCGATAAGAATTAGG

811 T S K S P K E N V V P P R K T S L D E L H F A F I P I R I R

2530 2540 2550 2560 2570 2580 2590 2600 2610

2521 AATCCGATCTCAAATGCAATTTCTGGAAGAAAAAATGTTAGTGATACTGAGAATGAAATCAGCCGAACGTTGCTGACAGCTGGGAAAAAT

841 N P I S N A I S G R K N V S D T E N E I S R T L L T A G K N

2620 2630 2640 2650 2660 2670 2680 2690 2700

2611 TATCTCAAGTCTGATCAGATACGCAGTGCAAGTAGTACAATAAAAAGTCATGTTCATTTGACAGTCATATCATTTATTTTAATGAAAGCA

871 Y L K S D Q I R S A S S T I K S H V H L T V I S F I L M K A

2710 2720 2730

2701 ACGATGTCAATTCATTTATGGAATATATTTTAA

901 T M S I H L W N I F *

Figure S2. Multiple alignment of the deduced amino acid sequences of *LAC1* from *B. tabaci* and its homologues from other insects. Conserved Cu-oxidase domains with identical amino acid residues are marked with black lines. Sequences with 100% homology are marked in black, sequences with ≥75% homology are marked in pink, and sequences with ≥50% homology are marked in blue. The origin of the *LAC* genes and their GenBank accession numbers are listed in Table S2.

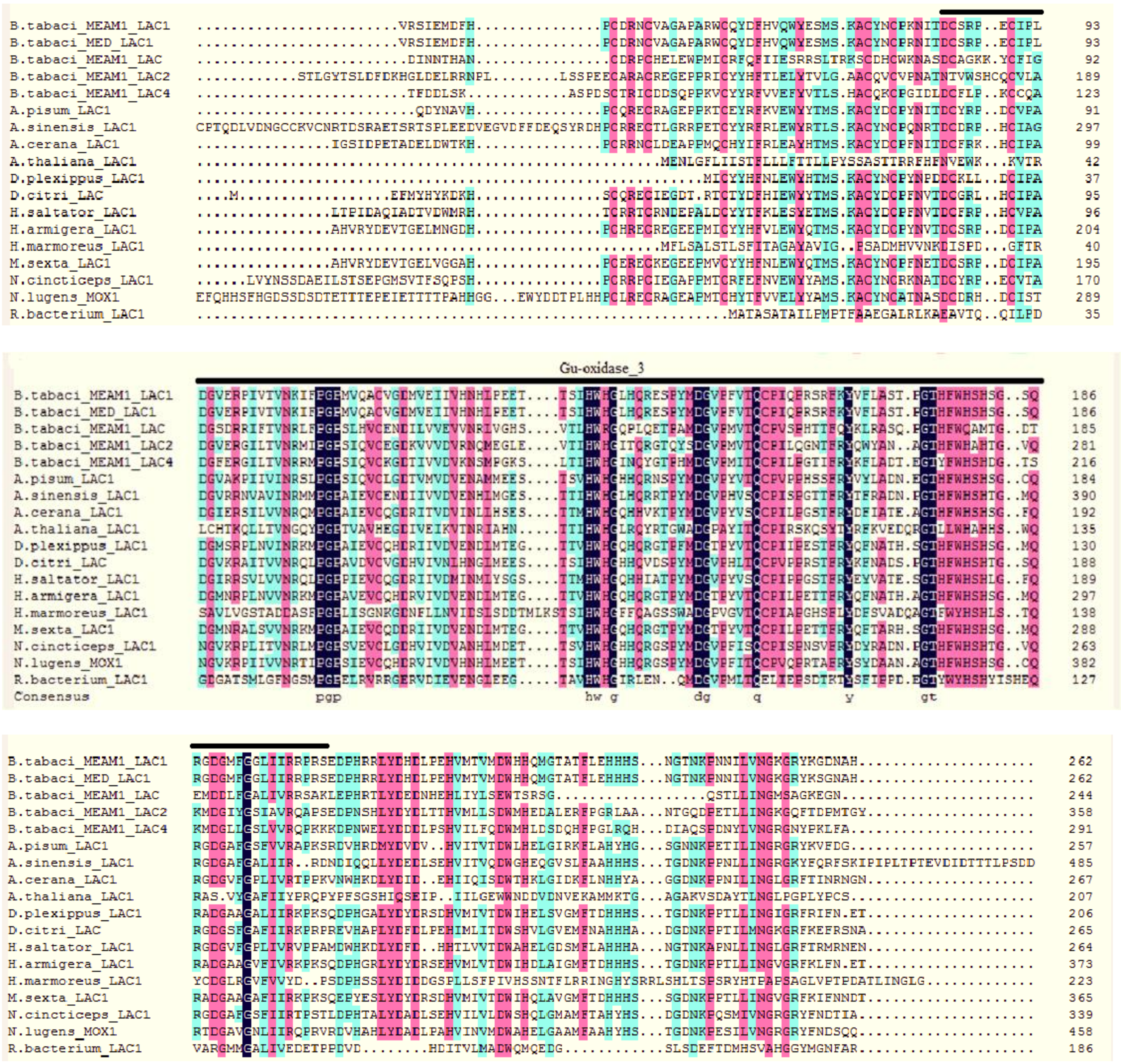

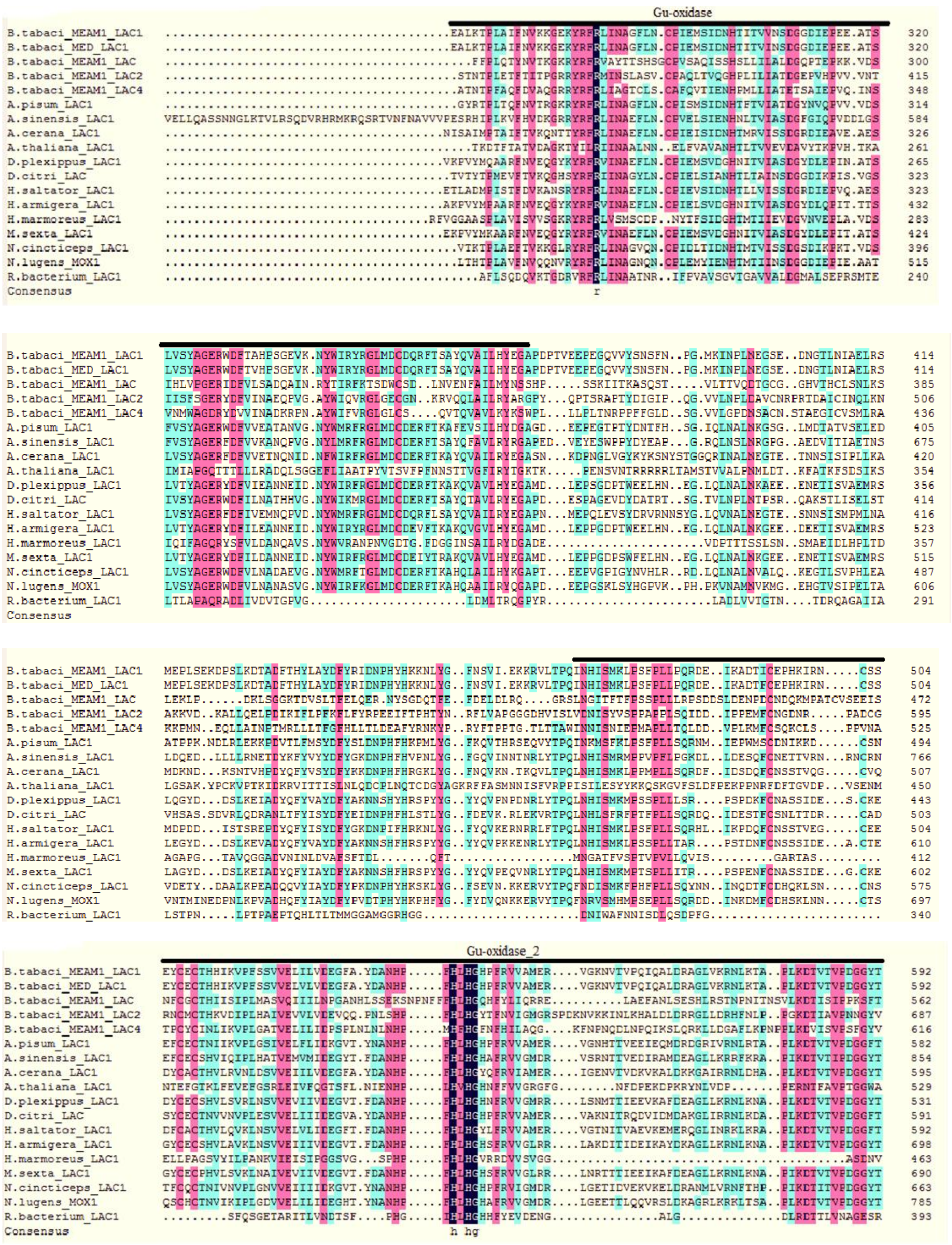
Gu-oxidase
Consensus
Gu-oxidase_2
Consensus

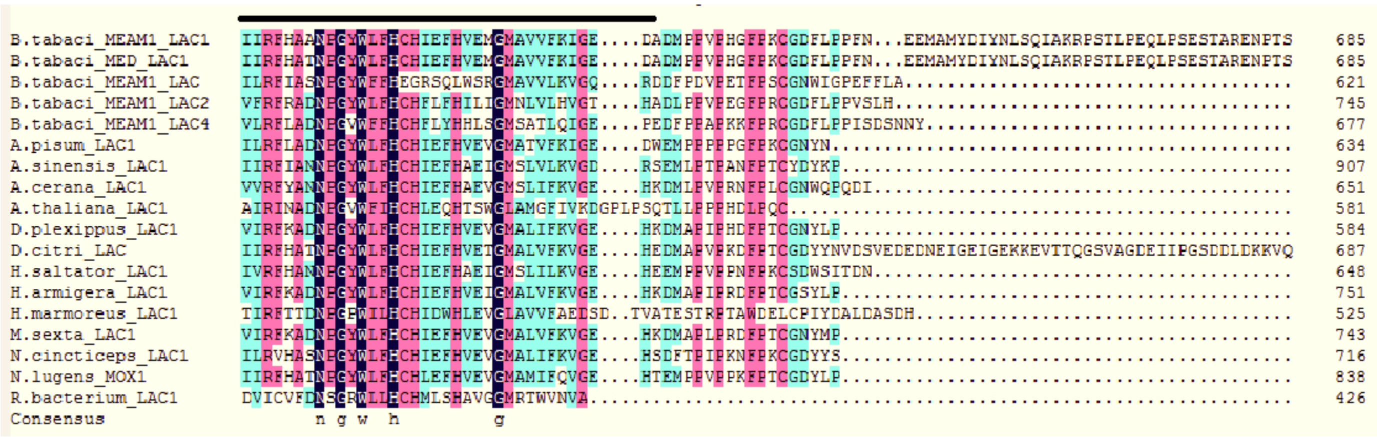

Table S1. Primer sequences used for cloning and quantitative real-time PCR

Experiments	Primer names and sequences(5′ to 3′)
Specific PCR	LF-TGACAAACGACTCTTCCGACC
	LR-ATTGTACTACTTGCACTGCGTATC
5′ RACE	NGSP1-AGTTGCAGGAGGCAAAGTGTAATCC
3′ RACE	NGSP2- CGAACGTTGCTGACAGCTGGGAAA
qPCR	Qlac1qF- ATTGCAGAGCTGAGGTCGAT
	Qlac1qR- ATGATTGATTTGCGGTGTCA
dsRNA	dsRNA F -TAATACGACTCACTATAGGG ACTCATTTCTGGCACTCGCA
	dsRNA R-TAATACGACTCACTATAGGGTCTTCCGGTTCGATGTCACC
dsGFP-F	dsGFP F-GGATCCTAATACGACTCACTATAGGTACGACTCACTATAGGAGTAAAGG
	dsGFP R-GGATCCTAATACGACTCACTATAGGTAGGTTTGTATAGTTCATCCATACC

Table S2. Sequences and relevant information used for alignment.

Species	GenBank No. or Gene ID.	Genes
B. tabaci MEAM1	AGC83693.1	*LAC1*
	Bta11878	*LAC*
	Bta11896	*LAC2*
	Bta00306	*LAC4*
Acyrthosiphon pisum	XP_001948070.1	*LAC1*
Anopheles sinensis	KFB43437.1	*LAC1*
Apis cerana	XP_016917075.1	*LAC1*
Arabidopsis thaliana	AEE29678.1	*LAC1*
Danaus plexippus	EHJ67706.1	*LAC1*
Diaphorina citri	XP_008487811.1	*LAC*
Harpegnathos saltator	EFN87217.1	*LAC1*
Helicoverpa armigera	AKH61981.1	*LAC1*
Hypsizygus marmoreus	AFN10626.1	*LAC1*
Manduca sexta	AAN17506.1	*LAC1*
Nephotettix cincticeps	BAJ06132.1	*LAC1*
Nilaparvata lugens	AKN21378.1	*MOX1*
Rhodobacteraceae bacterium HTCC2083	EDZ42302.1	*LAC1*

Table S3. Sequences and relevant information used for phylogenetic analysis

Species	Species Abbreviation	*Genes*	GenBank No. or Gene ID.
B. tabaci MEAM1	*BT MEAM1*	*LAC1*	AGC83693.1
		LAC	Bta11878
		LAC2	Bta11896
		LAC4	Bta00306
Acyrthosiphon pisum	*AP*	*LAC1*	XP_001948070.1
Anopheles gambiae	*AG*	*LAC1*	AAN17505.1
Anopheles sinensis	*AS*	*LAC1*	KFB43437.1
Apis cerana	*AC*	*LAC1*	XP_016917075.1
Apis mellifera	*AM*	*LAC1*	XP_001120790.2
Arabidopsis thaliana	*AT*	*LAC1*	AEE29678.1
Betula platyphylla	*BP*	*LAC1*	AKN79314.1
Bombus terrestris	*BT*	*LAC1*	XP_012163097.1
Coprinopsis cinerea	*CC*	*LAC1*	AAS38574.1
Coptotermes formosanus	*CF*	*LAC1*	AON96381.1
Coriolopsis trogii	*CT*	*LAC1*	AMJ39538.1
Danaus plexippus	*DP*	*LAC1*	EHJ67706.1
Daucus carota	*DC*	*LAC1*	ALR87190.1
Diaphorina citri	*DC*	*LAC*	XP_008487811.1
Harpegnathos saltator	*HS*	*LAC1*	EFN87217.1
Helicoverpa armigera	*HA*	*LAC1*	AKH61981.1
Hypsizygus marmoreus	*HM*	*LAC1*	AFN10626.1
Lenzites gibbosa	*LG*	*LAC1*	AEQ28163.1
Macrotermes barneyi	*MB*	*LAC*	AFD33366.1
Manduca.sexta	*MS*	*LAC1*	AAN17506.1
Neodiprion lecontei	*NL*	*LAC1*	XP_015512211.1
Nephotettix cincticeps	*NC*	*LAC1*	BAJ06132.1
Nilaparvata lugens	*NL*	*MOX1*	AKN21378.1
Operophtera brumata	*OB*	*LAC1*	KOB77133.1
Rhodobacteraceae bacterium HTCC2083	*RB*	*LAC1*	EDZ42302.1
Steccherinum murashkinskyi	*SM*	*LAC1*	AFI41888.1
Sulfitobacter guttiformis	*SG*	*LAC1*	KIN72709.1
Theobroma cacao	*TC*	*LAC1*	EOY21262.1
Trachymyrmex zeteki	*TZ*	*LAC1*	XP_018318119.1
Trametes versicolor	*TV*	*LAC1*	AAL00887.1
Volvariella volvacea	*VV*	*LAC1*	AGY56169.1
Zea mays	*ZM*	*LAC1*	AAX83113.1
Zootermopsis nevadensis	*ZN*	*LAC*	KDR15437.1
